# Opening of the Pulp Cavity and Preparation of the Roots for Filling

**Published:** 1874-03

**Authors:** Chas Kingsley


### DENTAL REGISTER.

VOL. XXVIII.] **MARCH, 1874.** [No. 3.

### OPENING OF THE PULP CAVITY AND PREPARATION OF THE ROOTS FOR FILLING.

BY CHAS. KINGSLEY.

*Read before the American Dental Society of Europe.*

The subject, as above expressed, to which I invite your attention presupposes the existence of one of two conditions:

First; where the pulp is still living, but conservative treatment is inadvisable, either on account of excessive inflammation, or partial loss of vitality: or second; where the pulp is not present in the tooth, or if present is devitalized. The causes of the first condition may be the irritation consequent upon the presence of caries in the tooth in proximity with the pulp: or from accident to the tooth externally, or to the parts surrounding the tooth. These are the most common causes, but not by any means the only ones. Let me say, however, that in the great majority of cases, the proximate cause is irritation consequent upon the pressure of caries, and the first step in all such cases is to remove the decayed matter from the tooth; more especially that portion in proximity or actual contact with the pulp. This should be done by first freely opening the cavity externally, by means of the enamel chisel; by the use of which it can be done with but slight pain, providing due care be exercised that the instrument does not slip too far into the cavity and cause pressure upon the pulp, or contiguous portions of the tooth.

In every case, the external opening of the cavity should be as nearly on a line with the axis of the root or roots of the tooth as is consistent with the strength of the tooth; thus avoiding, as much as possible, the entrance into the pulp canal at an angle. Having well opened the cavity externally, the next step is to remove the decayed or diseased dentine, in direct line with and actually covering the pulp. This need not be in ordinary cases an unusually painful affair, compared with the excavating of an ordinary cavity preparatory to filling. In the first place, let the operator choose a well-tempered, moderately small and keen-edged, hatchet excavator. The first portions of decay may be removed quite painlessly. As we approach the pulp, cut away the portions surrounding the exposed point of the pulp, or the point where the exposure is to be made, without at first exposing the pulp at its most accessible point; we shall then have but a small portion remaining in actual contact with the pulp, which can be removed with one or two well-directed and rapid strokes of the excavator. Burs or drills of any kind should not be used for this purpose.

Let us now suppose we are operating upon any one of the ten anterior teeth of either jaw, and have reached the point above indicated. The problem before us is, how to remove the pulp with the least injury to the vitality of the tooth, and surrounding parts, and at the same time with as little pain to the patient as possible; and here let me say, that I can not but express my deep regret, that many respectable practitioners of our specialty should have ever resorted to anything so unscientific, in view of its deleterious effects, as the use of arsenic in any form or quantity for devitalizing the dental pulp. Its deleterious effects are too well known to require repeating, and are frequently visible so long as the tooth in which it has been used remains in the mouth. This is especially the case when arsenic has been sealed up in the tooth and left to remain for any length of time; its use in this manner seems to me the greatest of the many crimes committed in the name of "Painless Dentistry." But to return. At this stage of the operation especially, and often from the beginning, the operator will find the rubber-dam of great assistance; not only to keep the tooth dry, under which conditions as a rule the operating can be done more rapidly and with less pain, but to protect the parts surrounding the tooth from the escarotics employed while removing the pulp and clearing the pulp canal.

The only instrument necessary for devitalizing and removing the pulp is a very fine, smooth pivot broach, such as are used by watch-makers. To prepare them for use the temper should be entirely drawn,—with especial care that the steel is not burned at the point,—and the broach thoroughly polished with moderately fine emery paper used *length-wise* of the broach. You have now an instrument almost as fine as a hair, with almost a perfect taper, and perfectly flexible. It can be forced through the pulp canal to the very apex of the root of the tooth,—however tortuous the pulp canal may be,—and with fair usage the danger of breaking off and remaining in the tooth is *nil.*

But in the mean time our pulp remains exposed, and from contact with the air is possibly aching. We should not have left it so long uncovered. Saturate the cavity with either creosote or pure carbolic acid,—(in some respects I prefer creosote, it being more powerfully escarotic,)—and introduce the broach into the pulp canal. In doing so conisder how slight is the union between the pulp and the walls of the pulp canal, and see that the instrument follows close to the side of the canal and does not at the outset force through, or into the pulp. Your instrument is so fine, that, except in the most constricted portion of the canal, it will impinge upon the pulp only sufficiently to give slight pain. But the pain may, to a great extent, be avoided by other means. The pulp canal with the broach in it may be compared to the parts of a pump, —the canal representing the barrel of the pump and the broach the piston. By working this piston back and forward, little by little, you pump the escarotic with which the cavity is charged into the pulp canal, and naturally, as in an ordinary pump, the fluid is forced a little in advance of the piston. If this process be continued, slowly advancing the piston little by little, the fluid can be pumped quite to the apex of the root, so much in advance of the instrument, that from the benumbing effects of the escarotic the pulp has lost much of its sensitiveness upon the approach of the instrument.

When the apex of the root is reached there must be an instant of pain, for the pulp can not be separated at this point from its connection with the parts exterior to the apicial foramen. But this is accomplished almost instantaneously by revolving the broach rapidly once or twice in the fingers. This need never fail to break the connection at once, unless there be an unusually large foramen as we sometimes find in the mouths of young subjects. In a case of this kind, withdraw the broach and slightly bend the point for a distance of 1-32 of an inch, and again introduce and carry it to the apex of the root. By revolving it now, you will be sure to completely amputate the pulp, and after running your instrument along the other sides of the canal to break up the union of the pulp with the walls of the canal on all sides, you are prepared to remove the devitalized pulp. This should be done at once and with the selfsame instrument,—by first winding around it some shreds of silk and leaving them to project for half an inch beyond the point,—then dipping it in creosote, carry it to the apex of the root once more, and after revolving it that the pulp may become entangled with the silk, withdraw it carefully, and after a few trials at most you will succeed in withdrawing the pulp twisted about your instrument.

After the pulp is removed, you have only to stop the hæmorrhage, and wipe out the canal, and it is ready for the gold. There are but very few cases of hæmorrhage after the extraction of the pulp which can not be stopped at once, by the application of creosote carried well up to the apex of the root and left there for a few moments. This can easily be accomplished by winding a little cotton upon the broach, pressing it well up the canal, and by turning the instrument backward one or two revolutions it will become disengaged from the cotton and can be removed, leaving the cotton and the creosote in the tooth. In cases of extreme inflammation and congestion of the pulp, there is more danger of the hæmorrhage being excessive, and in such cases it may sometimes be found advisable to leave the cotton and creosote in the tooth until another sitting. The wound caused by the removing the pulp in the manner I have described, heals quite as readily as any other wound of the same extent, and generally by first intention. I find as a rule that the healing process is not interfered with by the immediate filling of the root, except it be where the previous inflammation of the pulp has extended to some considerable extent to the parts beyond the apex of the root. In ordinary cases I prefer to complete the filling of the root at once, rather than leave it till a subsequent sitting. In either case there is felt a slight tenderness at the point of the root upon pressing upon the tooth, generally the second or third day after the operation. This indicates one step of the healing —the inflammatory—and rarely lasts but for one day.

Although I have in the foregoing specially mentioned the teeth anterior to the molars, still the method which I have described is equally applicable to any tooth of the entire set. I think the cases are very rare where treatment with arsenic is at all necessary—and wherever it is used it should be only as adjunct to the above described process, and never be left to remain in the tooth to become absorbed by it. In cases of extreme inflammation and hyper-sensitiveness of the pulp, arsenious acid or a mixture of it with sulphate of morphia and saturated with creosote may be applied to the pulp, instead of the pure creosote, and the operation continued in the ordinary manner. But I am convinced that a great deal of the pain consequent to the removal of the pulp can be avoided by a careful and skillful use of the instrument, nearly as much as in any ordinary surgical operation.

Where the nitrous oxide gas is at hand it is also very easy to make the operation entirely painless by its use; and the ultimate results as regards the tooth is fully worth all the trouble ef administering the gas to amputate the pulp instantaneously, rather than deteriorate it by the use of arsenic. Let us forever hereafter leave the use of this drug to that legion of charlatans which hang on the skirts of our profession, and are at the same time the curse of suffering humanity and our shame.

We have now to consider the second part of our subject according to the classification made,—i. e.—the preparation of so-called dead teeth. The preliminary steps do not vary materially from those described in the first part of this essay,—except that, there being no living tissue in the pulp canal, the same care is not required to avoid giving pain. Before, however, the pulp canal is in condition for filling, it must be perfectly cleansed of all dead or decayed matter,—the entire root disinfected, and all inflammation subdued.

How is this best accomplished ?

In the treatment of teeth of this class, we are frequently called upon to counteract and correct the deleterious results of other men's carelessness, incapacity or dishonesty. The great majority of teeth of this class that come under our professional treatment, have previously been in the hands of others of our professional brethren, and their condition as we find them is the direct result of the treatment they then received. We must not, however, attach blame to those cases where honest and careful conservative treatment of the exposed pulp has resulted in an ultimate failure, as in our present degree of enlightenment will sometimes occur. In the majority of cases no such treatment has been attempted, and frequently we find where the practitioner has confined his efforts to filling the cavity in the tooth, after having devitalized the pulp, but without removing the dead tissue from the pulp canal, but left it fully impregnated with arsenic to act upon the bony tissue of the tooth and the surrounding parts. Again, in other cases, the pulp has been carefully removed, but the operator not having filled the pulp canal, infiltration has occurred through the apicial foramen, and we find a condition, if not so bad as the last, still likely to result in alveolar abscess. The difficulty of treating teeth of this class is in proportion to the quantity and character of the diseased matter in the pulp canal, and to the length of time which it has been retained there to impregnate the bony tissue of the tooth. We often find that although the tooth has remained in this condition for years, without giving serious trouble, but slowly disintegrating and waiting for a slight provocation to crumble to pieces,—still when we attempt to remove this diseased mass which has lain comparatively inert for so long a time active symptoms of inflammation of the periosteum supervene. To avoid this sometimes requires extremely careful and patient treatment. The first efforts to remove the dead matter from the canal should be confined to opening the pulp cavity, and syringing it with tepid water. After an interval of one or two days we may commence to apply antiseptics, and to more completely remove by mechanical means the dead matter from the tooth. I find the same plain broach, of a larger size however, the most valuable instrument of all for this purpose. With it the dead matter can be broken up and dislodged from its place, and readily washed out with the syringe, or wiped out with a little cotton or silk wound upon the broach. Great care should always be exercised that the instrument does not pass through the foramen and wound even to the slightest extent, the parts beyond,—otherwise, we may be called upon to confront symptoms not unlike those of a dissecting or other poisoned wound. Care should also be taken that the antiseptics employed at first do not act too violently, causing irritation and inflammation. Generally it is safer to commence with spirits of camphor, eau de cologne or carbolic acid diluted with three or four parts of water. As the treatment progresses, more powerful disinfecting agents may be employed, and usually the third or fourth application may be of pure carbolic acid or creosote. This should be applied on cotton, and the cavity sealed up for two or three days, (sometimes but one) that the antiseptic may become thoroughly absorbed by the tooth; at the end of which time the cotton may be removed and the root filled without the danger of future trouble.

The most simple cases of all to treat are those where the pulp has died without treatment or inflammation of the surrounding tissues, but has not yet decomposed. These cases rarely require more than to remove the dead pulp and wipe out the canal with creosote, before filling. These cases, however, are rarely met with.

I am convinced that, with a majority of my professional brethren, this subject has not received its due attention. Certainly there is not a more important subject of consideration to be met with in the practice of our specialty, and so it will remain to be until the day comes when we shall be able to preserve the life of the pulp under all conditions to fulfill its functions of nourishment of the tooth.

Alveolar abscess is a condition too often met with in the mouths of our patients. Let us hope that the members of our profession will give more attention to the prevention of this great cause of suffering.

